# A Phase Ib/II open-label study to evaluate the safety and efficacy of MEDI-551 in combination with immunomodulating therapy in patients with relapsed or refractory aggressive B cell lymphomas

**DOI:** 10.1186/2051-1426-2-S3-P73

**Published:** 2014-11-06

**Authors:** Trishna Goswami, Paola Canelos, Radhika Parikh

**Affiliations:** 1 MedImmune, USA

## Background

MEDI-551, an IgG1k antibody-dependent cellular cytotoxicity (ADCC) enhanced anti-CD19 monoclonal antibody (mAb), has a single-agent response rate of 24% (12% with complete remission [CR]) in heavily pretreated patients with diffuse large B cell lymphoma (DLBCL). MEDI0680 (AMP-514) a humanized IgG4κ mAb against PD-1, blocks inhibitory PD-1 receptors (PD-L1) on T cells to augment immune responses. A combination of MEDI-551/MEDI0680 (AMP-514) may link the intrinsic cytotoxic capability of MEDI-551 with an agent that primes the immune cells required for ADCC or may augment immune responses from tumor cell death by MEDI-551. This study explores this novel combination regimen in patients with multiply relapsed/refractory (R/R) DLBCL who have limited opportunities for cure, and may offer a regimen with less toxicity but equal or better efficacy versus traditional chemotherapy. Study D2852C00004, a Phase Ib/2 dose escalation/expansion study, will determine the maximum tolerated dose (MTD) or the highest protocol-defined dose (HPDD) safety, and activity of the combination of MEDI-551/MEDI0680 (AMP-514) in R/R DLBCL patients who have failed 1-2 prior lines of therapy.

## Study objectives

Primary objectives: to determine the MTD or HPDD (in the absence of exceeding the MTD) of MEDI-551/MEDI0680 (AMP-514); dose expansion: to evaluate the safety, tolerability, and clinical activity of MEDI-551/MEDI0680 (AMP-514). Secondary objective: to evaluate the pharmacokinetics and antidrug antibodies of MEDI-551/MEDI0680 (AMP-514) in patients with R/R DLBCL. Exploratory objectives will determine the impact of tumor PD-L1 expression on clinical activity of the combination and will follow soluble PD-1 and PD-L1 expression as biomarkers of activity.

## Study design

Standard 3+3 design to determine the MTD of the combination of MEDI-551/MEDI0680 (AMP-514). Patients will receive MEDI-551 12 mg/kg once monthly with 1 of 3 possible doses of MEDI0680 administered bimonthly for 1 year. Thereafter, patients will continue MEDI-551 monthly until progressive disease or toxicity. If a patient experiences progressive disease beyond 12 months of therapy while still receiving MEDI-551 treatment, combination treatment with MEDI0680 (AMP-514) may be resumed for up to 12 additional months. After MTD identification, additional patients will be enrolled in the dose expansion portion to ensure a total sample size of 26 efficacy-evaluable patients. Thirteen of 26 efficacy-evaluable patients receiving the MTD will be required to have PD-L1 expression. Patients will be stratified by refractoriness to chemotherapy, and CD20 therapy and PD-L1 status. Results will inform additional studies to find a chemotherapy-free regimen for frail R/R DLBCL patients.

